# Characterisation of Invasive *Streptococcus pneumoniae* Isolated from Cambodian Children between 2007 – 2012

**DOI:** 10.1371/journal.pone.0159358

**Published:** 2016-07-22

**Authors:** Catrin E. Moore, Adam Giess, Sona Soeng, Poda Sar, Varun Kumar, Pheakdey Nhoung, Rachel Bousfield, Paul Turner, Nicole Stoesser, Nicholas P. J. Day, Christopher M. Parry

**Affiliations:** 1 Mahidol-Oxford Tropical Medicine Research Unit, Faculty of Tropical Medicine, Mahidol University, Bangkok, Thailand; 2 Angkor Hospital for Children, Siem Reap, Cambodia; 3 Nuffield Department of Medicine, University of Oxford, Oxford, United Kingdom; 4 Centre for Tropical Medicine and Global Health, Nuffield Department of Medicine, University of Oxford, Oxford, United Kingdom; 5 Microbiology Department, Addenbrooke’s Hospital, Cambridge, United Kingdom; 6 Cambodia-Oxford Medical Research Unit, Angkor Hospital for Children, Siem Reap, Cambodia; 7 Clinical Research Department, London School of Hygiene and Tropical Medicine, London, United Kingdom; 8 School of Tropical Medicine and Global Health, Nagasaki University, Nagasaki, Japan; Instituto Butantan, BRAZIL

## Abstract

**Background:**

The 13-valent pneumococcal vaccine (PCV13) was introduced in Cambodia in January 2015. There are limited data concerning the common serotypes causing invasive pneumococcal disease (IPD). Knowledge of the circulating pneumococcal serotypes is important to monitor epidemiological changes before and after vaccine implementation.

**Methods:**

All episodes of IPD defined by the isolation of *Streptococcus pneumoniae* from blood, cerebrospinal fluid or other sterile site in Cambodian children admitted to the Angkor Hospital for Children in Siem Reap, Northwestern Cambodia, between 1^st^ January 2007 and 1^st^ July 2012 were retrospectively studied. *Streptococcus pneumoniae* isolates that could be retrieved underwent phenotypic typing and whole genome sequencing.

**Results:**

There were 90 Cambodian children hospitalized with IPD with a median (IQR) age of 2.3 years (0.9–6.2). The case fatality was 15.6% (95% CI 8–23). Of 50 *Streptococcus pneumoniae* isolates available for further testing, 46% were penicillin non-susceptible and 8% were ceftriaxone non-susceptible, 78% were cotrimoxazole resistant, 30% were erythromycin resistant and 30% chloramphenicol resistant. There were no significant changes in resistance levels over the five-year period. The most common serotypes were 1 (11/50; 22%), 23F (8/50; 16%), 14 (6/50; 12%), 5 (5/50; 10%) and 19A (3/50; 6%). Coverage by PCV7, PCV10 and PCV13 was 44%, 76% and 92% respectively. We identified novel multilocus sequence types and resistotypes using whole genome sequencing.

**Conclusions:**

This study suggests IPD is an important disease in Cambodian children and can have a significant mortality. PCV13 coverage of the serotypes determined in studied strains was high and consistent with another recent study. The phenotypic resistance patterns observed were similar to other regional studies. The use of whole genome sequencing in the present study provides additional typing and resistance information together with the description of novel sequence types and resistotypes.

## Introduction

*Streptococcus pneumoniae* (pneumococcus, *S*. *pneumoniae*) causes an estimated one million childhood deaths each year, mostly in developing countries [1]. *S*. *pneumoniae* is carried in the upper airways of healthy individuals, particularly infants and children and is easily transmitted between household members [2]. Strains carried in the upper airways are considered the reservoir of isolates that may cause lower respiratory tract infections, pneumonia and invasive disease. The polysaccharide capsule is an important virulence factor of the pneumococcus and the distribution of the >90 different capsular serotypes varies geographically and over time [3]. The serotypes causing disease in Asia have been recently reviewed but data is limited for Cambodia [3–6]. In a recent study of *S*. *pneumoniae*, 88% of 43 invasive isolates serotypes causing invasive pediatric disease in Cambodia in 2013–14 had a serotype covered by the PCV13 vaccine [7].

Studies conducted by the Global Alliance for Vaccines and Immunization (GAVI Alliance) and the Pneumonia Etiology Research Child Health Project (PERCH) are contributing to our understanding of childhood pneumonia and associated diseases in Asia but have not so far included Cambodia [8]. Pneumococcal conjugate vaccines (PCV) active against 7, 10 or 13 of the most common disease-causing serotypes of *S*. *pneumoniae* are now being deployed in these areas [9]. Data from countries surrounding Cambodia such as Laos, Thailand and Vietnam show that the PCV7 has coverage of 39–53%, PCV10 coverage of 34–78% and PCV13 coverage of 45–81% of cases [3, 8, 10–15]. Knowledge of pneumococcal serotypes from invasive disease in the pediatric population of Cambodia will support the Cambodian public health authorities make informed decisions about vaccination implementation. Monitoring of the prevailing serotypes will also be important to observe potential serotype replacement.

In Cambodia, and other developing countries, where access to healthcare is limited and unregulated drug prescribing is commonplace, antimicrobial resistance is increasing [5, 16]. It is important to define the extent of resistance to the antimicrobials in common use in each country against important pathogens to inform antimicrobial guidelines both locally and countrywide. The high level of antimicrobial resistance in these countries is worrying and supports the argument for pneumococcal vaccination.

We report on invasive pneumococcal disease in children attending a children’s hospital in Siem Reap, Northwestern Cambodia between 2007 and 2012, and describe the antibiotic resistance profiles, serotypes, vaccine coverage and multilocus sequence types on a subset of isolates. This study complements and extends the data from the 2013–4 surveillance study [7].

## Materials and Methods

### Hospital setting

During the study period, Angkor Hospital for Children (AHC) was a 50-bedded charitably funded pediatric hospital in Siem Reap, Northwestern Cambodia. The hospital provides free medical care for children <16 years of age, with approximately 125,000 attendances and 4,000 admissions each year over the study period, however the hospital has since grown to >80 beds, the current available treatments are described in more detail online (http://angkorhospital.org/treatment/). *S*. *pneumoniae* causes approximately 10% of bloodstream infections in this hospital [17].

### Patients studied

Children admitted to AHC who had *S*. *pneumoniae* cultured from a sterile site (blood, cerebrospinal fluid (CSF), joint fluid or other sterile site), between 1^st^ January 2007 and 1^st^ July 2012 were defined as having invasive pneumococcal disease (IPD). Cases were identified from the Microbiology Department laboratory records. Demographic and clinical data were retrieved from the hospital electronic database.

### Microbiology methods

Sterile body fluids were processed using standard microbiological methods. *S*. *pneumoniae* isolates were identified by colony morphology, lack of catalase reaction, Gram stain, susceptibility to optochin and bile solubility. Antimicrobial susceptibility was determined for all available strains by disk diffusion to oxacillin, co-trimoxazole, ceftriaxone, erythromycin and chloramphenicol, and by E-test (Biomerieux, France) to penicillin, co-trimoxazole and ceftriaxone in accordance with CLSI guidelines [18]. Isolates were stored at -80°C in Tryptone Soya Broth (TSB) with 10% glycerol and transported to Oxford for further testing where serotyping was performed using the Quellung reaction.

### Whole genome sequencing

Whole Genome Sequencing (WGS) was performed to confirm the capsule serotype, determine multilocus sequence type (MLST) (http://spneumoniae.mlst.net) and resistotype *in silico*. Reads were mapped to the serotype 6B resistant *S*. *pneumoniae* reference genome (RefSeq: NC_014498) isolated in Spain using a validated in-house bioinformatics pipeline [19]. The *de novo* reads were assembled using Velvet to determine the different capsule types [20]. EBURST version 3 was used to determine the relationships with regards to MLST types [21]. The resistance genes previously described (*folA*, *folP*, *pbp1A*, *pbp2B*, *pbp2X*) were identified using a nucmer search for the flanking region using the aligned and assembled DNA as previously described [22–25]. Where the gene could not be identified from the aligned DNA the *de novo* gene assembly was interrogated for the resistance gene. Each gene was aligned and translated in Geneious v8.1.6 (Biomatters Ltd, US) and the resistance markers were documented.

### Data analysis

The data were analysed using STATA v13.1 (StataCorp, Texas, USA).

### Ethical permission

The Institutional Review Board at AHC and the Oxford Tropical Research Ethics Committee (506–12) gave ethical approval for this retrospective study. As the study was retrospective in nature, spanning a five-year period, consent was not obtained from each patient when the study was conceived in 2012. All patient information was anonymised and de-identified during the laboratory work. The final analysis was performed on data that was non-identifiable.

## Results

### Patient characteristics

There were 90 confirmed IPD patient episodes during the study period (78 isolates from blood, 4 CSF, 8 other sterile fluids). The median (IQR, range) age of the children was 2.3 years (0.9–6.2; 1 day-14.5 years) with 37 (41.1%) under two years of age, 25 (27.8%) two to four years of age and 28 (31.1%) over five years. Forty (44.4%) patients were female. The final clinical diagnosis given by the responsible clinician was severe pneumonia in 47 (52.2%) patients, bacteremia in 16 (17.8%), septic shock in 12 (13.3%), meningitis in eight (8.9%), cellulitis in two (2.2%), pyomyositis in one (1.1%), unrelated infections in two (1.1% each, one urinary tract infection and one dengue infection) and no diagnosis recorded in two (2.2%). Thirteen children died in hospital and one was discharged moribund to die at home, giving a case fatality rate of 15.6% (95% CI 8–23). Ten of the 62 (16.0%) children <5-years old died (95% CI 6.7–25.5).

### Quellung serotypes

**Fig 1:**
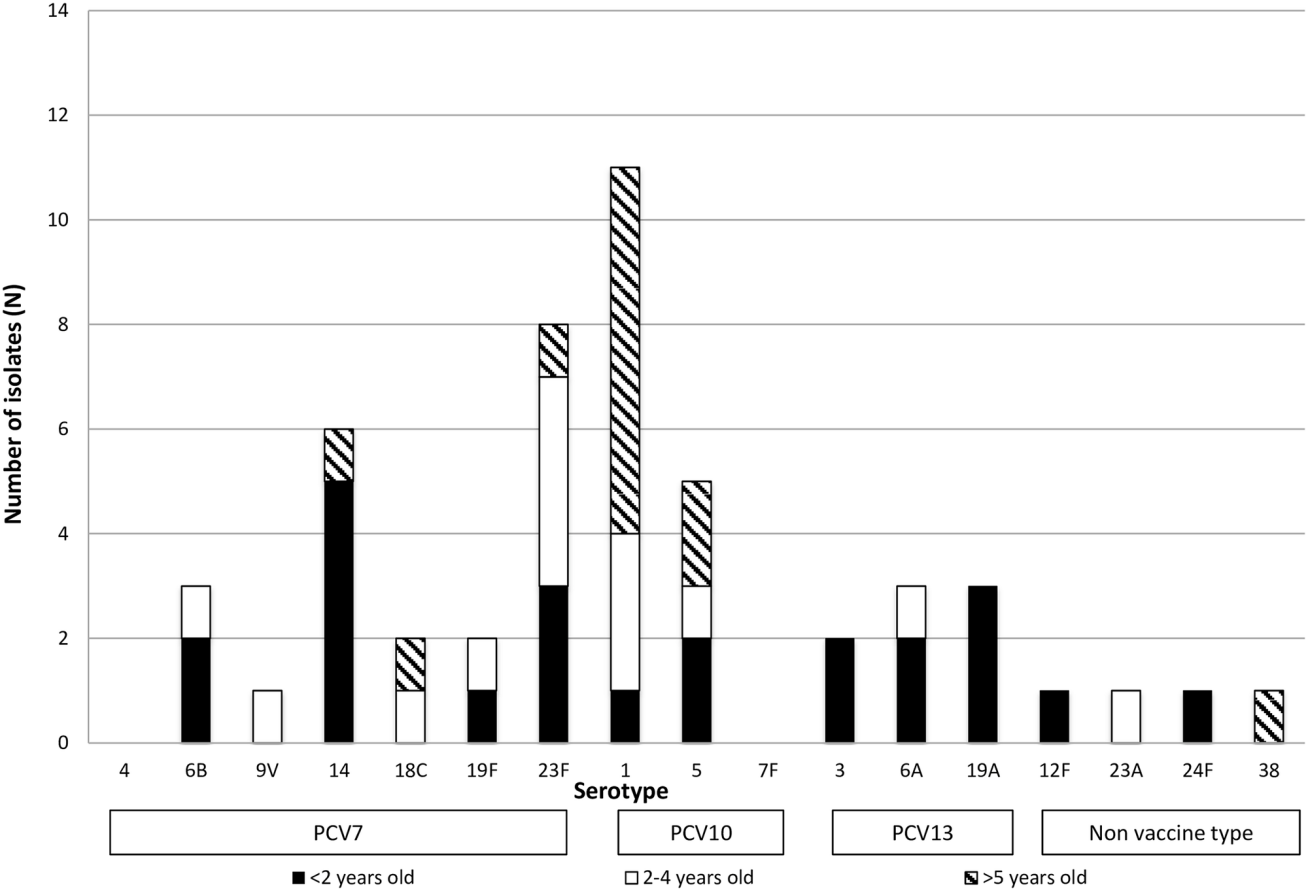
Serotype results by age and vaccine coverage.

There were 50 strains that were viable when sub-cultured and available for further testing. Among the 50 strains there were 16 different serotypes (S1 Table together with year of isolation). The five most frequent serotypes for all ages were serotypes 1 (n = 11 strains, 22%), 23F (n = 8, 16%), 14 (n = 6, 12%), 5 (n = 5, 10%), 19A (n = 3, 6%) 6A (n = 3, 6%) and 6B (n = 3, 6%) (Fig 1). The PCV7 vaccine (4, 6B, 9V, 14, 18C, 19F and 23F) would cover 22 of the cases (44%; 95% CI 30–58); PCV10 (PCV7 with additional serotypes 1, 5 and 7F) would cover 38 cases (76%, 95% CI 64–88); and PCV13 (PCV10 plus 3, 6A and 19A) would cover 46 cases (92%; 95% CI 84–100), including 21 infections in children under two (91%; 95% CI 78–100). Overall, four (8.0%) strains were not covered by the current vaccines.

### MLST sequence types

We determined 23 different sequence types (STs) with seven (13.5%) new STs (two for serotype 23F and one each for 18C, 23A, 6B, 19F and 3) (Table 1). The most numerous STs were ST217 (n = 11) that were all serotype 1, ST289 (n = 5) all serotype 5, ST782 (n = 5) all serotype 14, ST9050 (n = 4) all serotype 23F and ST320 (n = 3) all serotype 19A.

**Table 1:** MLST, serotyping and resistotyping data for invasive *S*. *pneumoniae* isolates.

Serotype	MLST CC	MLST ST	Number, n = 50 (%)	DHFS variant^#^	DHPS* variant	Co-trimoxazole MIC result		Functional penicillin single nucleotide polymorphisms (SNPs; pbp1A|pbp2B|pbp2X)	Penicillin MIC result	
						Susceptible	Resistant		Susceptible	Non-susceptible
1	217	217	11 (100)	EEHMKPIAQV**A**E**I**KPHFA	STRPRPGSSYVEIE	0	11	NSQFLAGSFSYLTYTMAAGVIPTAV	10	1
14	63	782	5 (83.3)	**EDHMKPIAYVADLKPHFA**	STRPRPGSSYVEIE	0	2	NSQFLAGSFSYLTYATAAGVIPTAV	0	2
					STRPRPGSSYVEIE	0	3	NSQFLAGMPSYLTYATAAGVIPTAV	0	3
	63	63	1 (16.7)	EDYMKSITYV**A**D**L**KPHFA	STRPRPGSSYVEIE	0	1	NSQFLAGSFSYLTYTTAAGVIPTAV	0	1
23F	9050	9050	4 (50)	EDYMKSIVHI**G**D**L**KSHST	STRPRPGSSYVEIE	0	1	NSQFLASFPSQRFGTTAAGVIPTAV	0	1
					STRPRPGSSYVEIE	0	3	NSQFLAGSFSYLTYTTAAGVIPTAV	1	2
	81	81	1 (12.5)	DDHMKSLAHV**A**D**L**KPHST	STRPGSSSYVEIE	0	1	GTGYMVGSF-YLTYATSSSYLTSVI^¶^	0	1
		242	1 (12.5)	EDHMKSLAHV**A**E**L**KPQYK	STRPRPGSSYVEIE	0	1	GTGYMVGSFSYLTYATSSSYLTSVI	1	0
		new ST1	1 (12.5)	EEHMKPIAQV**A**E**I**KPHFA	STRPRPGSSYVEIE	0	1	NSQFLAGSFSYLTYTTAAGVIPTAV	1	0
		new ST2	1 (12.5)	EEHMKPIAQV**A**E**L**KPHFA	STRPRPGSSYVEIE	0	1	GTGYMVGPMSYLTYTTAAGVIPTAV	1	0
5	**289**	**289**	**5 (100)**	**EEHMKPIAQVAEIKAHFA**	**STRPGSSYVEIE**	**4**	**0**	**NSQFLAGSFSYLTYTMAAGVIPTAV**	**4**	**0**
					**STRPGSSYVEIEIE**	**1**	**0**	**NSQFLAGSFSYLTYTMAAGVIPTAV**	**1**	**0**
19A	320	320	3 (100)	EDYMKSITYV**A**D**L**KPQFT	STRPGSSYSYVEIE	0	1	GTGYMVGSFSYLTYATSSSYLTSVI	0	1
					STRPGSSYVEIEIE	0	1	GTGYMVGSFSYLTYATSSSYLTSVI	0	1
					STRPGSSYVEIEIE	0	1	ITGYMVGSFSYLTYATSSSYLTSVM	0	1
6B	156	95	2 (66)	EDHMKLIAHV**V**E**L**KSHFA	STRPGRSSYVEIE	0	2	GTGYMVGSFSYLTYATSSSYLTSVI	0	2
	156	new ST	1 (33)	EEHMKPIAQV**A**E**I**KPHFA	STRPGRSSYVEIE	0	1	NSQFLAGSFSYLTYTTAAGVIPTAV	1	0
9V	**156**	**280**	**1 (100)**	**EEHMKPIAQVDEIKPHFA**	**STRPGSSYVEIE**	**1**	**0**	**NSQFLAGSFSYLTYTMAAGVIPTAV**	**1**	**0**
24F	**156**	**5549**	**1 (100)**	**EEHMKPIAQVAEIKAHFA**	**STRPGSSYVEIE**	**1**	**0**	**NSQFLAGSFSYLTYATAAGVIPTAV**	**1**	**0**
6A	Singleton	5421	2 (67)	EDHMKSLAHV**A**E**L**KPQYS	STRPGSSYGYVEIE^§^	0	2	NTGYMVGSFSYLTYTTAAGVIPTAV	0	2
		incomplete^$^	1 (33)	EDHIKPITYV**D**D**L**KPHFA	STRPGSSYSYVEIE	0	1	GTGYMVGSFSYLTYTTAAGVIPTAV	0	1
18C	**3594**	**3594**	**1 (50)**	**EEHMKPIAQVAEIKPHFA**	**STRPGSSYVEIE**	**1**	**0**	**NSQFLAGSFSYLTYTMAAGVIPTAV**	**1**	**0**
		new ST	1 (50)	EDHMKPIAQV**A**E**I**KPHFA	STRPGRSSYVEIE	0	1	NSQFLAGSFSYLTYTMAAGVIPTAV	1	0
3	4909	4909	1 (50)	EEHMKPIAQV**A**E**I**KPHFA	STRPGSSYVEIE	1	0	NSQFLAGSFSYLTYTMAAGVIPTAV	0	1
		**new ST**	**1 (50)**	**EEHMKPIAQVAEIKPHFA**	**STRPGSSYVEIE**	**1**	**0**	**NSQFLAGSFSYLTYTMAAGVIPTAV**	**1**	**0**
19F	Singleton	new ST	1 (50)	EDHMKLIAHV**V**E**L**KSHFA	STRPGSSYYVEIE^§^	0	1	GTGYMVGSFSYLTYTTAAGVIPTAV	0	1
		Incomplete^$^	1 (50)	DDHMKSLAHV**A**D**L**KPHST	STRPGSSYYVEIE^§^	0	1	GTGYMVGSFSYLTYTTAAGVIPTAV	0	1
38		**310**	**1 (100)**	**EEHMKPIAQVAEIKPHFA**	**STRPGSSYVEIE**	**1**	**0**	**NSQFLAGSFSYLTYTMAAGVIPTAV**	**1**	**0**
23A	Singleton	new ST	1 (100)	EKHMKPIAQV**A**E**L**KAHFA	STRPRPGSSYVEIE	0	1	NSQFLAGSFSYLTYTMAAGVIPTAV	1	0
12F	989	989	1 (100)	EKHMKPIAQV**A**E**I**KAHFA	STRPGRSSYVEIE	0	1	NSQFLAGSFSYLTYTMAAGVIPTAV	1	0

All variants labeled in bold indicate strains sensitive to both co-trimoxazole and penicillin, MLST CC = Multilocus sequence type clonal complex, MLST ST = MLST sequence type

^#^Dihydrofolate reductase (DHFR), represents amino acid residues 14–149 in the amino acid sequence of DHFR from *S*. *pneumoniae* R6, the amino acid in bold represent residues 92 and 100

*Dihydropteroate synthase (DHFS), Represents amino acid residues 56–67 in the amino acid sequence of DHPS from *S*. *pneumoniae* R6

^§^Three variants have not previously been reported

^¶^One strain was not fully typed for penicillin resistance

^$^All seven MLST alleles unavailable for these strains

### Antibiotic resistance and resistotyping

The MIC_50_, MIC_90_ (range, interquartile range) for penicillin MIC in 50 isolates was 0.06μg/ml, 0.50μg/ml (0.03–2.0μg/ml, 0.12–0.25μg/ml). All isolates were penicillin susceptible using the recently revised CLSI pneumonia breakpoints (susceptible ≤2 μg/ml) [18]. By the meningitis breakpoints, which are often applied in epidemiological analyses and where penicillin non-susceptibility is indicated by an MIC of >0.06μg/ml [18], 46% of strains were non-susceptible to penicillin and in three strains the penicillin MIC was ≥1mg/ml (Table 2). Forty-six (92%) isolates were susceptible to ceftriaxone by meningitis breakpoints with four intermediate strains (MICs of 0.5 μg/ml (n = 2), and 1.0μg/ml (n = 2)), which were also non-susceptible to penicillin (MICs of 0.5 μg/ml, 1.0μg/ml (n = 2) and 1.5μg/ml). One strain was serotype 19F (MICs 0.5 μg/ml), one 23F (MICs of 1.0 μg/ml), and two 19A (MLST ST320), which are covered by PCV13. When categorized by clinical syndrome, one strain causing meningitis was penicillin non-susceptible (MIC 0.096 μg/ml) using the CLSI meningitis breakpoints but no strains were ceftriaxone non-susceptible (Tables 2 and 3) [18]. Resistance to cotrimoxazole was found in 78% of strains and to erythromycin and chloramphenicol independently in 30% of strains. There were 39 (78%) strains resistant to at least one antimicrobial and 23 (46.0%) displayed resistance to three or more drugs, including known resistant serotypes 23F (n = 6; STs 81, 242, 9050 and a new ST), 14 (n = 5; STs 63 and 782) and 19A (n = 3; ST 320).

**Table 2:** Antibiotic susceptibility of invasive *S*. *pneumoniae* isolates overall and by age of child.

Antimicrobial	Number of isolates (%) susceptible						
	Under 2 years n = 24 (%)	2–4 years n = 13 (%)	5 years and over n = 13 (%)	All patients n = 50 (%)	Susceptible	Intermediate	Resistant
Penicillin							
All strains—non-meningitis breakpoint^1^	24 (100)	13 (100)	13 (100)	50 (100)	≤ 2	4	≥ 8
All strains—meningitis breakpoint^2^	9 (38)	8 (62)	10 (77)	27 (54)	≤ 0.06	-	≥ 0.12
Non-meningitis strains^1^	22/22 (100)	13/13 (100)	13/13 (100)	48/48 (100)	≤ 2	4	≥ 8
Meningitis strains^2^	1/2 (50)	0	0	1/2 (50)	≤ 0.06	-	≥ 0.12
Ceftriaxone							
All strains—non-meningitis breakpoint^1^	23 (96)	13 (100)	13 (100)	49 (98)	≤ 1	2	≥ 4
All strains—meningitis breakpoint^2^	20 (83)	13 (100)	13 (100)	46 (92)	≤ 0.5	1	≥ 2
Non-meningitis strains^1^	22/22 (100)	13/13 (100)	13/13 (100)	48/48 (100)	≤ 1	2	≥ 4
Meningitis strains^2^	2/2 (100)	0	0	2/2 (100)	≤ 0.5	1	≥ 2
Erythromycin^3^	15 (63)	8 (62)	12 (92)	35 (70)	≥ 21	16–20	≤ 15^2^
Co-trimoxazole	6 (25)	2 (15)	3 (23)	11 (22)	≤ 0.5/9.5	1/19-2/38	≥ 4/76
Chloramphenicol^3^	21 (88)	8 (62)	6 (46)	35 (70)	≥ 21	-	< 20

^1^ Susceptibility determined using the CLSI non-meningitis breakpoints with penicillin non-susceptibility indicated by an MIC of >4μg/ml as per CLSI guidelines

^2^ Susceptibility determined using the CLSI meningitis breakpoints with penicillin non-susceptibility indicated by an MIC of >0.06μg/ml as per CLSI guidelines

^3^ Antibiotic disc testing rather than MIC as per CLSI guidelines

**Table 3:** Minimum inhibitory concentrations (MICs) for penicillin and ceftriaxone by disease.

Disease	Total number	Samples examined for MIC	Penicillin MIC			Penicillin category^1^ n (%)			Ceftriaxone MIC			Ceftriaxone category^1^ n(%)		
			MIC_50_	MIC_90_	Range	S	I	R	MIC_50_	MIC_90_	Range	S	I	R
Pneumonia	47 (52.2)	28 (56.0)	0.016	0.50	0.006–1.00	28	0	0	0.047	0.38	0.004–0.75	28	0	0
Bacteremia	16 (17.8)	10 (20.0)	0.008	0.94	0.002–1.50	10	0	0	0.064	0.75	0.003–1.00	10	0	0
Septic shock	12 (13.3)	5 (10.0)	0.016	0.38	0.012–0.38	5	0	0	0.016	0.38	0.012–0.38	5	0	0
Meningitis	8 (8.9)	2 (4.0)	0.008	0.051	0.008–0.094	1	0	1	0.030	0.047	0.012–0.047	2	0	0
Cellulitis	2 (2.2)	1 (2.0)	0.016	-	-	1	-	-	0.012	-	-	1	-	-
Pyomyositis	1 (1.1)	0	-	-	-	-	-	-	-	-	-	-	-	-
Uncertain site	4 (4.4)	4 (8.0)	0.056	0.75	0.012–0.75	4	0	0	0.072	0.50	0.012–0.50	4	0	0
Total	90	50	0.06	0.5	0.03–2.0	49	0	1	0.05	0.5	0.03–1.00	50	0	0

^1^ Penicillin parenteral and ceftriaxone categorization using non-meningitis or meningitis breakpoints according to the site of infection as per CLSI guidelines,. S: susceptible, I: intermediate; R: resistant

Genotyping for known functional polymorphisms causing penicillin and co-trimoxazole resistance are in Table 1. Ten different penicillin determinants were observed with the most common (genotype: NSQFLAGSFSYLTYTMAAGVIPTAV) found in 46.2% of isolates, for which all of the penicillin and ceftriaxone MICs were <0.096 μg/ml. The second most common genotype (at 14%) included the two ceftriaxone intermediate strains and all strains were resistant to penicillin (genotype: GTGYMVGSFSYLTYATSSSYLTSVI). We also analyzed the *folA* (dihydrofolate reductase [DHFR]) and *folP* (dihydropteroate synthase [DHPS]) sequences of the isolates. In the DHFR enzyme all the resistant isolates had a substitution of leucine for iso-leucine at position 100. Eight co-trimoxazole resistance variants were observed in *folP* with a 6bp insertion leading to the insertion of two additional amino acids in DHPS the most common [23]. Three of the co-trimoxazole variants in the current study have not previously been reported. Two DHFS patterns were observed for the strains susceptible to co-trimoxazole, they mainly differed at one base from those resistant (amino acid 111; alanine in the sensitive strains and proline in the resistant strains).

## Discussion

In this study of 90 Cambodian children admitted to hospital with IPD, almost half of the children were under two years of age and one in six died. The study isolates were collected during the period before the start of PCV13 introduction. The range of pneumococcal serotypes determined in the available isolates was consistent with those in surrounding countries and with a study conducted in 2013–14 at the same centre [2, 5–7, 15, 26–28]. PCV13 was introduced into the national programme given as a 3+0 dosing schedule with no additional catch-up campaign, with GAVI Alliance support, in January 2015 [29]. Our data suggests that the PCV13 will potentially protect against the organisms causing 92% of IPD (95% CI 84%-99%) in these children over this five-year period. In the study conducted in 2013–14, coverage by PCV13 was 88% for invasive strains and 63% in the colonizing strains (62–71%) [7].

The STs of serotype 1 (ST217), serotype 14 (ST63/784), and serotype 5 (ST289) strains were similar to those found elsewhere. The serotype data was similar to the 2013–14 study with serotype 1 the most numerous, other serotypes were less common with the difference between the studies difficult to evaluate [7, 30]. More than 10% of the other STs in this study were novel. The number of multi-drug resistant (MDR, resistant to > 3 classes of drugs) invasive strains was 46%, slightly lower than the level of 56% found in the subsequent study, although the second study examined additional antibiotics [7]. The detection of serotypes 19A (ST320) and 23F (ST 9050) in Cambodia is interesting because of the potential for resistance gene transfer to other strains in the same niche due to the high rate of recombination in *S*. *pneumoniae*. Strains of the MDR resistant serotype 19A (ST320) have emerged in a number of Asian countries [31, 32]. Both 19A and 23F are covered by PCV13, therefore introduction of the vaccine may lead to a decrease in antibiotic resistance in the *S*. *pneumoniae* strains circulating in this region, although subsequently the NVTs described in Turner *et al*. were also resistant [7, 31, 32].

Penicillin non-susceptibility using CLSI breakpoints according to the clinical syndrome, was present in nearly half of the strains tested with two strains of intermediate susceptibility to ceftriaxone using the CLSI meningitis breakpoints. All isolates were penicillin susceptible using the recently revised CLSI pneumonia breakpoints. Two thirds of strains were resistant to co-trimoxazole consistent with the later local study [7]. The levels of resistance are broadly similar to those in surrounding areas, which range in resistance levels from 33% (both intermediate and fully resistant) in the Philippines to 99% in India [33]. The proportion of strains resistant to erythromycin was the same as invasive isolates in the later local study [7], similar to one study in the region [34], but lower than others [35, 36]. Resistance is likely to be driven by the overuse of antibiotics in the community. Antibiotics can be obtained without prescription or the need for a medical consultation in Cambodia and may be given at sub-therapeutic doses and for a shorter period than recommended. Our recent study documented that one third of patients attending AHC out-patients had evidence of recent antibiotic use, and in addition use of sub-standard antibiotics is likely to be widespread [37, 38]. Drug quality in developing countries is difficult to measure and drug regulations can be difficult to enforce, leading to a large market in low-quality antimicrobials, in turn contributing to an increase in antimicrobial resistance of bacteria in the region.

With increasing use of WGS of clinical isolates a greater variety of resistance genotypes for different antibiotics are likely to be identified [22]. The penicillin genotypes in this study have been described in more detail elsewhere and no single pattern was associated with non-susceptibility [23]. A large number of the strains showed phenotypic resistance to co-trimoxazole and WGS identified eight different genotypes for the DHFS and seventeen for the DHFR [39]. A single genotype accounted for the majority of the susceptible Cambodian strains (DHFS: STRPGSSYVEIE and DHPS: EEHMKPIAQVAEIKAHFA), this genotype was not restricted to serotype/ST (present in serotypes 5, 9V, 24F, 18C, 3 and 38). All of the resistant strains contained the amino acid leucine at position 100 substituted in place of isoleucine in DHFS [40]. Thirty one (62%) of the co-trimoxazole resistant strains contained the 6bp insertion in DHPS and eight contained a 3bp insertion that has also been described in Malawi [23]. Three novel co-trimoxazole resistance genotypes are described in the current work including one genotype (two strains) that contained a 3bp insertion and another that contained a novel 6bp insertion. Although co-trimoxazole resistance is high in Cambodia, current use of the drug is mainly for treating patients with melioidosis or in those infected with HIV.

This study has a number of limitations including the retrospective design and relatively the small numbers of IPD cases. Reassuringly the results are broadly consistent with a subsequent prospective study at the same site [7]. Only a sub-set of the isolates was available for further testing because a number of strains could not be re-grown when sub-cultured. This limited the analysis of serotype distribution and antibiotic resistance patterns. The gradual improvement of laboratory capacity which has increased the isolation rates in the present study, together with the improved awareness of the utility of microbiology by local clinicians since 2005 contributed to the improved blood culture yield observed in the 2013–14 study. Antibiotic pre-treatment in the community before hospital admission is common and may have biased the isolates toward more resistant strains. Data from this single site may not be generalizable to the whole of Cambodia and data from other sites are needed.

## Conclusions

This study covering the period 2007 to 2012 confirms and extends the 2013–14 data from the same centre, the use of whole genome sequencing in the present study provided additional typing and resistance information with the description of novel sequence types and resistotypes. It highlights a high mortality associated with invasive pneumococcal disease in hospitalized Cambodian children and the potential benefit of the PCV13 vaccination program. It emphasizes the need for detailed prospective studies to describe the serotypes of carriage and invasive pneumococci circulating in Cambodia after vaccine introduction.

## Supporting Information

S1 Table The *S*. *pneumoniae* serotypes detected and year of isolation. (DOCX)
