# Supplementary material for: Characterisation of Invasive Streptococcus pneumoniae Isolated from Cambodian Children between 2007 – 2012
Source: PLoS One. 2016 Jul 22;11(7):e0159358. doi: 10.1371/journal.pone.0159358 (PMC4957771; doi:10.1371/journal.pone.0159358)
Supplement: S1 Table — (DOCX) [file pone.0159358.s001.docx]

|  | Year | | | | | |  |
| --- | --- | --- | --- | --- | --- | --- | --- |
| Serotype | **2007** | **2008** | **2009** | **2010** | **2011** | **2012** | **TOTAL** |
| 1 | 0 | 0 | 1 | 4 | 3 | 3 | **11** |
| 3 | 0 | 0 | 0 | 1 | 0 | 1 | **2** |
| 5 | 0 | 0 | 0 | 0 | 5 | 0 | **5** |
| 6A | 0 | 0 | 0 | 0 | 2 | 1 | **3** |
| 6B | 0 | 0 | 0 | 1 | 1 | 1 | **3** |
| 9V | 0 | 0 | 0 | 1 | 0 | 0 | **1** |
| 12F | 0 | 0 | 0 | 0 | 0 | 1 | **1** |
| 14 | 2 | 0 | 1 | 1 | 2 | 0 | **6** |
| 18C | 0 | 0 | 0 | 1 | 0 | 1 | **2** |
| 19A | 0 | 1 | 0 | 1 | 1 | 0 | **3** |
| 19F | 0 | 1 | 0 | 0 | 0 | 1 | **2** |
| 23A | 0 | 0 | 0 | 0 | 0 | 1 | **1** |
| 23F | 1 | 1 | 2 | 0 | 3 | 1 | **8** |
| 24F | 0 | 0 | 0 | 0 | 1 | 0 | **1** |
| 38 | 0 | 0 | 0 | 1 | 0 | 0 | **1** |
| Total | 3 | 3 | 4 | 11 | 18 | 11 | 50 |
